# Internal cost of spontaneous deception revealed by ERPs and EEG spectral perturbations

**DOI:** 10.1038/s41598-019-41962-z

**Published:** 2019-04-01

**Authors:** Chengkang Zhu, Jingjing Pan, Shuaiqi Li, Xiaoli Liu, Pengcheng Wang, Jianbiao Li

**Affiliations:** 1 China Academy of Corporate Governance, Business School, Nankai University, Tianjin, China; 2 School of Economics, Shandong University, Jinan, China; 3 Reinhard Selten Laboratory, Nankai University, Tianjin, China; 4 Nankai University Binhai College, Tianjin, China; 5 Business School, Tianjin University of Economic and Finance, Tianjin, China

## Abstract

Abundant literature has studied the behavioral and neural correlates of deception, but little research has focused on the internal cost of spontaneous deception. In the present study, the event-related potential and event-related spectral perturbations techniques were used to measure the internal cost of spontaneous deception by having participants perform a sender–receiver task in which they decided whether to send deceptive messages to increase their payoff from the task. Several important main findings emerged from this study. We observed a reward positivity (RewP) after senders sent the message, suggesting an integration of reward with associated cost after response in our task. Furthermore, spontaneous deception decreased the amplitude of the RewP and power in the delta and beta bands, whereas it increased the amplitude of power in the theta band, indicating that deception carried an internal cost that devalued individuals’ rewards.

## Introduction

In contrast with the classic approach in economics, which assumes that people are selfish and that lying by itself does not carry any cost, accumulated evidence corroborates that in some cases, people are averse to lying in economic interactions. From a psychological perspective, people internalize social norms that serve as an internal value system^1,2^. Noncompliance gives rise to internal cost^3^. As lying is against the social norm, when deciding whether to lie, people might face an internal trade-off between honesty to avoid the potential internal cost of lying and dishonesty for personal material gain^4^. Evidence in support of the potential positive internal costs of lying primarily derives from behavior experiments, in which several players are unwilling to lie even at the expense of earning extra money^3,5,6^.

Although many behavior experiments have indicated that people opt for honesty with the intention of averting possible internal punishment, little is known about whether lying carries any internal cost for individuals and devalues their rewards when they make the decision spontaneously^4,7–9^. A previous study proposed a self-concept maintenance hypothesis in which cheaters consistently find a magnitude of dishonesty with which they derive certain financial benefits from dishonest behavior yet maintain a positive self-concept in terms of honesty^3^. If this is the case, spontaneous lying would not cause any internal cost, but if it is not the case, the reward that comes from spontaneous lying would be devalued by the concomitant internal cost^3,5,6^. However, to our knowledge, compelling evidence that supports/rejects such a hypothesis is lacking.

Existing neuroscientific studies have focused on the neural processes that underlie instructed and spontaneous deception^10–14^. Previous research using functional magnetic resonance imaging (fMRI) reported stronger activity in the prefrontal cortex (PFC) and right temporo-parietal junction (rTPJ). The subjects were instructed to make dishonest statements (i.e., possession of an item, personal information or experience, knowledge of mock crime, and valence of picture) compared with honest statements^15–18^. By contrast, spontaneously limited honest behavior was associated with neural activity in brain regions related to cognitive control, such as the anterior cingulate cortex (ACC), right dorsolateral prefrontal cortex (rDLPFC), and ventrolateral prefrontal cortex (VLPFC)^19^. One fMRI study confirmed a significant difference in neural activity patterns between instructed and spontaneous lying^20^. In the study, subjects guessed the sum of a dice roll and bet either “big” or “small.” After learning the result, the subjects reported whether their bet was correct. The subjects’ reports, rather than the prediction results, determined their final payoff. In the spontaneous session, the participants could freely make the decision. Meanwhile, in the instructed session, the participants were instructed to report their betting results honestly or dishonestly according to randomly selected instructions. The results of the two sessions were compared. The authors affirmed that instructed decisions did not elicit similar activation patterns in certain regions, such as the subgenual ACC, DLPFC, VLPFC, and inferior parietal lobule, which were sensitive to either spontaneous truth-telling or lying. Casual approaches using transcranial direct-current stimulation provided further evidence for the involvement of the rDLPFC and rTPJ in spontaneous decision making^21–23^.

With a millisecond-level resolution, the event-related potential (ERP) and event-related spectral perturbations (ERSP) techniques are used to probe the neural dynamics of lying. Certain literature using ERP has studied the cognitive process involved in instructed lying and found enhanced cognitive negative variation, P300, and late positive components and potentials for lying compared with truth-telling. This notion indicates that lying requires high levels of effortful involvement and cognitive load^24–27^. Nevertheless, the participants in these studies were explicitly instructed by an experimenter to make untruthful statements in which they would not materially benefit from lying. Thus, the participants neither genuinely decided to be honest nor faced a trade-off between honest behavior and material gain. The subjects’ electronic potentials, which indicated high levels of effortful involvement and cognitive load, could not represent the existence of the internal cost of spontaneous deception. Only one ERP study has investigated cheating in a setting that involved a moral trade-off between honesty and financial gain^28^. However, the study instructed the participants to maintain a rate of lying of up to 50% and did not focus on the internal cost of lying.

At the electrophysiology level, electroencephalogram (EEG) markers related to reward consumption after deceptive decision making could be used to measure the internal cost of spontaneous deception. Reward processing can be decomposed into two phases: motivation to chase a reward (i.e., decision making and reward anticipation) and reward consumption (i.e., outcome evaluation and hedonic pleasure)^29,30^. Performance monitoring (PM), a complex ability to rapidly evaluate performance outcomes and detect errors, has been traditionally related to reward consumption. During reward consumption, PM exploits specific EEG signals representing the integration of reward with associated cost in both the time and time-frequency domains^31^.

In the time domain, the ERP component reward positivity (RewP) could capture the rapid integration of reward with cost^32^. The RewP peaks at approximately 300 ms at channels Fz or FCz after a performance outcome is known. The amplitude of RewP corelates with the value of an option that decreases as a function of associated cost. In early research, the RewP was not found for a time since it was always suppressed by the negativity related to the expectation (NE) in the experimental task (i.e., door task)^32^ and was viewed as feedback-related negativity (FRN)^33–37^. In a task in which players knew the information about their payoff after feedback, FRN peaking at 250–350 ms at the frontocentral sites after feedback delivery was influenced by not only the reward evaluation but also the reward prediction^36,38–42^. A recent study confirmed that the neural systems giving rise to the RewP and FRN were partly dissociable^43^. To extract the RewP, some researchers used principle component analysis (PCA), a data reduction technique that helps to disentangle overlapping ERPs (i.e., RewP and NE)^32^.

Integration processing also influences non-phase-locked EEG oscillation in the time-frequency domain^31^. The power of parietal delta band (1–4 Hz) activity, the primary time-frequency component underlying the RewP, is sensitive to reward evaluation and could be an index representing the integration of reward with associated cost in the time-frequency domain^31,44–49^. Another prominent component during reward processing is theta band (4–7 Hz) activity. Theta power might reflect loss, whereas delta power reflects gain in the door task^32,48–50^. Moreover, theta band activity is associated with cognitive control or cognitive effort^44,51–54^. Finally, in the beta band (12–30 Hz) at central and frontal midline sites, increased power has also been associated with reward evaluation^51,55,56^. Furthermore, beta power is sensitive to reward expectations and reward magnitude^55,57^.

**Figure 1:**
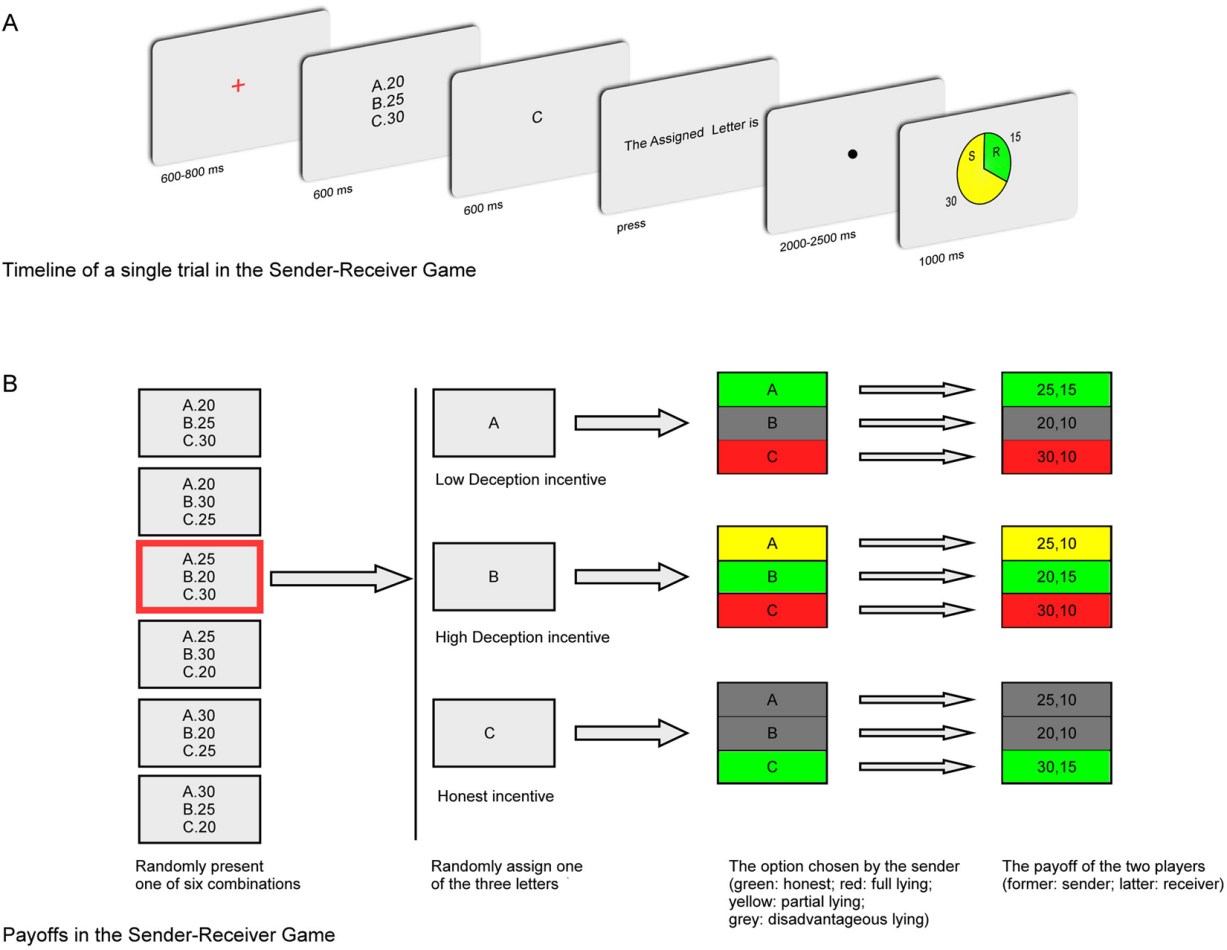
Overview of the task and trial structure. (**A**) At the beginning of each trial, three letters (i.e., **A**–**C**) with different payoffs (i.e., 20, 25, and 30) were presented. After one of these letters appeared randomly, the sender chose an option to send a message about this letter to the receiver and earned the payoff associated with his/her chosen option. (**B**) According to the associated payoff for the assigned letter, there were three conditions, i.e., HI, LDI and HDI. Then, the sender’s actions were divided into four categories, i.e., honest, full lying, partial lying and disadvantageous lying, depicted by green, red, yellow and gray, respectively. The sender’s payoff depended only on his/her chosen option, and the receiver’s payoff was dependent on whether the sender sent the true message.

The present study primarily aimed to investigate the internal cost of spontaneous deception. To this end, we conducted an ERP experiment with a total of 45 participants who played a two-player sender–receiver game paradigm that was adopted from previous behavioral studies and modified for the current experiment^4^ (Fig. 1). In the experiment, the main unit of analysis was defined as a “trial,” in which two players were referred to as the “sender” and the “receiver”. In each trial, three options (i.e., A, B, C) with different possible payoffs (i.e., 20, 25, 30) were shown on the screen. The computer then randomly assigned a letter A, B or C to the sender. Subsequently, the sender chose an option to send a message about this letter to the receiver. The sender’s payoff was in accordance with his/her chosen option and independent of the receiver’s decision. For example, if the sender chose option A, and the payoff for option A was 30, then he/she would earn 30 CNY for this trial. The receiver’s payoff was dependent on whether the letter he/she selected was the same letter as that assigned to the sender. If so, the receiver would earn 15 CNY for this trial; otherwise, he/she would earn 10 CNY.

In the game, the sender’s payoff, which was independent of the receiver’s action, relied only on the message he/she sent to the receiver, whereas the receiver’s payoff was dependent on whether his/her own choice was correct. This paradigm overcame the drawback of strategic motives (i.e., senders may tell the truth to induce receivers to take the wrong action if the senders assume that their partner will not follow their recommendation). For this reason, senders would balance the certain material reward and cost of lying only when they decided whether to lie. Moreover, since senders knew their payoff as soon as they made choices, the integration of reward with its corresponding cost would occur after the choice was made instead of after feedback onset. Thus, this procedure enabled us to separate reward evaluation from reward prediction error detection so that we could elicit a relatively pure RewP and its underlying time-frequency components linked to the integration process in this task. We expected to observe an obvious RewP at approximately 300 ms at FCz or Cz after the senders sent the message. By using PCA, we also expected to obtain an obvious PCA-RewP from 300 ms to 400 ms at Fz or FCz. As illustrated in Fig. 1, we specifically established three conditions, i.e., honest incentive (HI), low deception incentive (LDI) and high deception incentive (HDI). The payoff for senders sending truthful messages in the HI condition was identical to that for senders sending fully deceptive messages that maximized their payoffs in the LDI and HDI conditions. Under the self-image maintenance hypothesis, we expected that (i) in the time domain, the amplitude of the RewP and PCA-RewP would not be modulated by deception and (ii) in the time-frequency domain, the power of the delta band, theta band and beta band would be comparable between deception and truth-telling when controlling senders’ payoff. Otherwise, if spontaneous deception in fact carries an internal cost for participants, when controlling senders’ payoff, we expected that (i) in the time domain, truth-telling would elicit a larger amplitude of the RewP and PCA-RewP than deception and (ii) in the time-frequency domain, compared with deception, truth-telling would elicit larger power in the delta band and beta band but smaller power in the theta band.

## Results

### Behavior data

**Figure 2:**
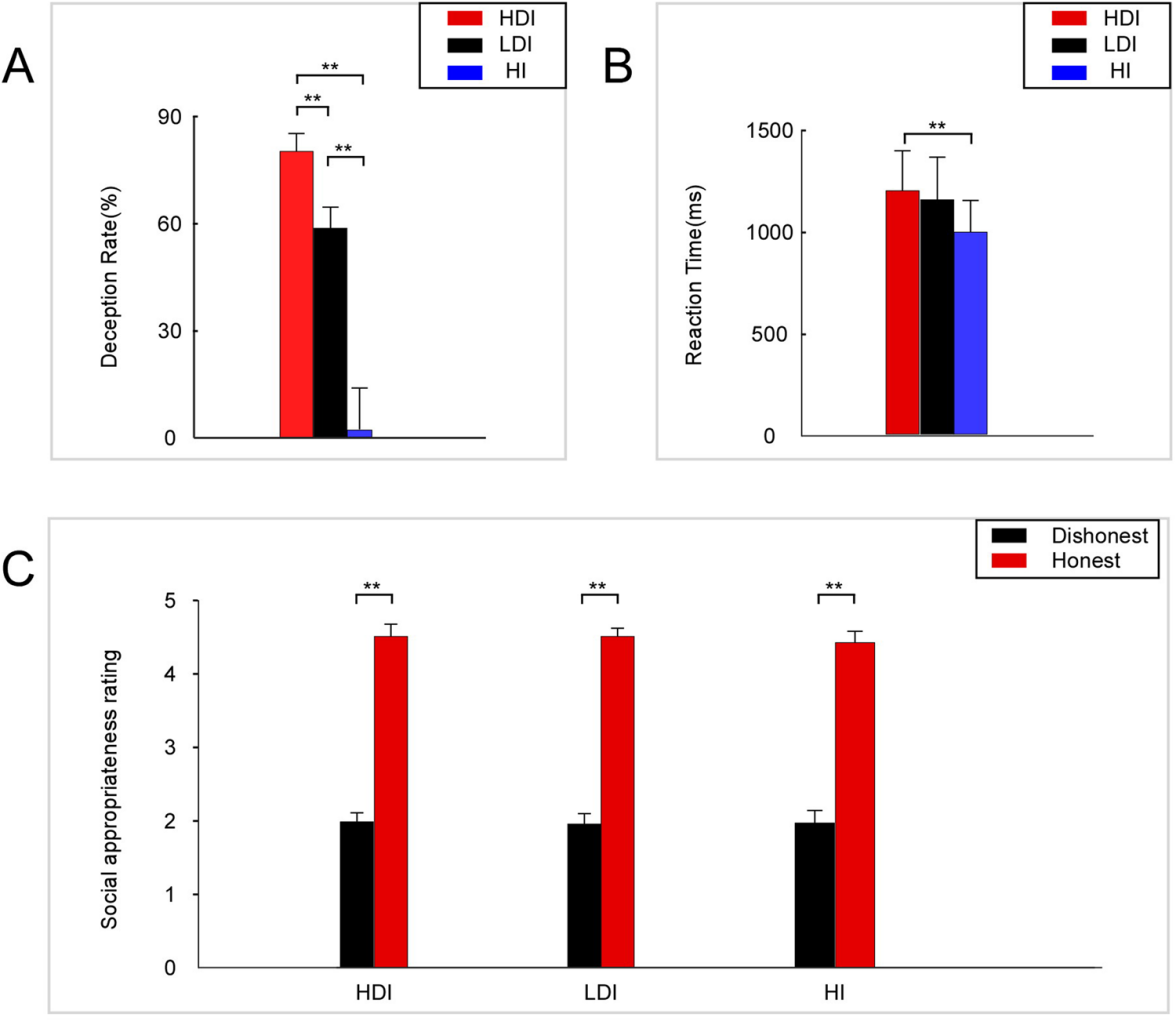
Behavioral results. (**A**) Deception rates among conditions (HI, LDI, and HDI). (**B**) Reaction time among conditions (HI, LDI and HDI). (**C**) Social appropriateness rating for honest vs. dishonest responses among conditions (HI, LDI and HDI). Error bars represent s.e.m. *p < 0.1, **p < 0.05.

For all the conditions, 48.43% of messages were true. The frequency with which the senders deceptively revealed the message was defined as the deception rate. The deception rate was analyzed using one-way repeated measure ANOVAs (rmANOVAs) with conditions (HI, LDI, and HDI). A significant effect [F (2,88) = 115.417, p < 0.001, partial η^2^ = 0.724, N = 45] was found, with a higher deception rate for the HDI condition (mean ± se, 0.83 ± 0.05) than for the LDI condition (mean ± se, 0.61 ± 0.06, p < 0.001) and HI condition (mean ± se, 0.02 ± 0.012, p < 0.001), as well as for the LDI condition compared with the HI condition (p < 0.001) (see Fig. 2A).

Reaction time was analyzed using one-way rmANOVAs with conditions (HI, LDI, and HDI). Significant effects of condition [F (2,88) = 5.653, p = 0.011, partial η^2^ = 0.114, N = 45] were found, with a higher reaction time for the HDI condition (mean ± se, 1,169.49 ± 192.14 ms) than the HI condition (mean ± se, 1,126.33 ± 204.79 ms, p = 0.005) (see Fig. 2B).

In each condition, the mean score of the deceptive actions rated by each participant was defined as the social appropriateness rating of dishonest response. The social appropriateness ratings were analyzed using two-way rmANOVAs with response (honest vs. dishonest) and conditions (HI, LDI, and HDI) as factors. A significant main effect was found for response [F (1,44) = 268.10, p < 0.001, partial η^2^ = 0.859, N = 45] with a larger score for the honest response (mean ± se, 4.511 ± 0.09) than the dishonest response (mean ± se, 1.970 ± 0.107) (see Fig. 2C). However, neither a significant main effect for condition nor a significant interaction effect was found (for details, see Supplementary Table S1).

### Electrophysiological data

#### Time domain analysis

**Figure 3:**
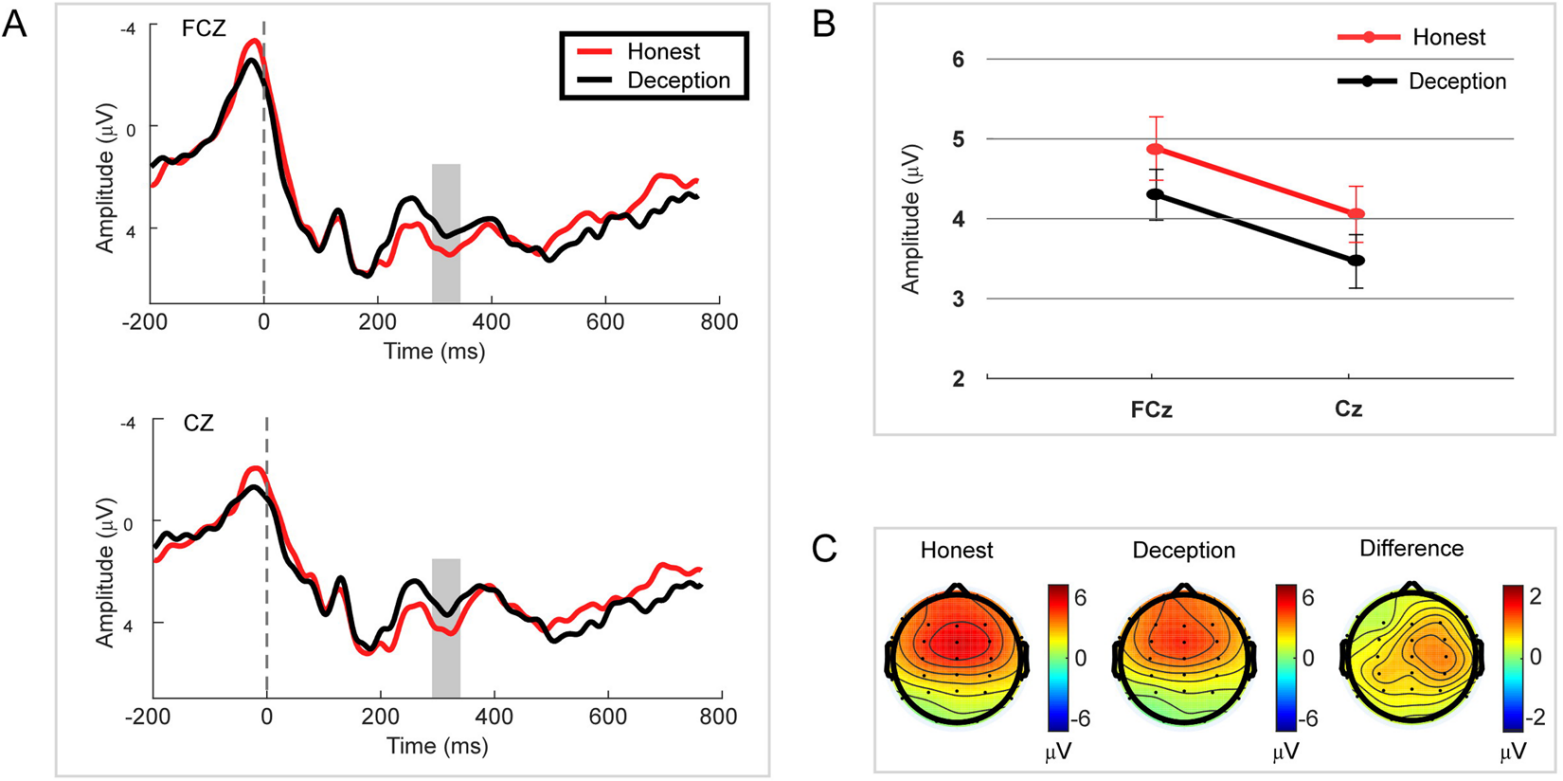
RewP results. (**A**) Grand average ERP waves computed at a set of midline sites (FCz and Cz). (**B**) Mean amplitude of the RewP at two sites for Honest vs. Deception responses showing two significant main effects for both response and site. (**C**) Topographic voltage maps of mean amplitude of the RewP.

We assessed the ERPs evoked by Honest and Deception responses. The RewP was observed to be evident at approximately 330 ms after Honest and Deception responses at a set of midline sites (FCz, Cz). We submitted a 2 × 2 rmANOVA with response (Honest vs. Deception) and site (FCz vs. Cz) as factors. A significant main effect was found for response [F (1,37) = 10.592, p = 0.002, partial η^2^ = 0.223, N = 38] with a larger amplitude for the Honest response (mean ± se, 4.559 ± 0.356 μV) than for the Deception response (mean ± se, 3.730 ± 0.338 μV). Moreover, a significant main effect was also found for site [F (1,37) = 13.544, p = 0.001, partial η^2^ = 0.268] with a larger amplitude at FCz (mean ± se, 4.436 ± 0.355 μV) than at Cz (mean ± se, 3.852 ± 0.309 μV) (see Fig. 3). However, insignificant interaction effect was found.

**Figure 4:**
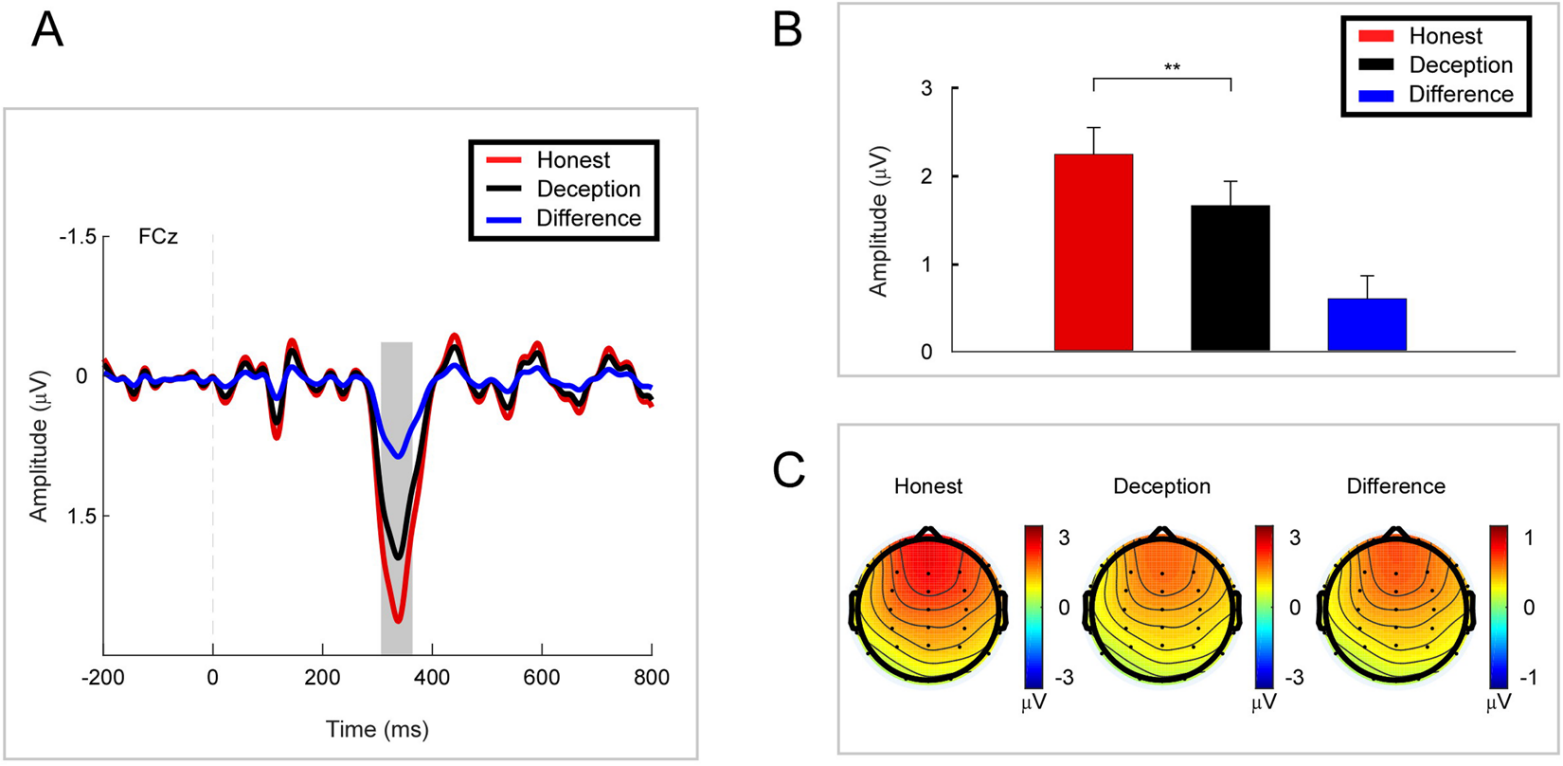
PCA-RewP results. (**A**) Grand average ERP waves computed at Fz. (**B**) Mean amplitude of the PCA-RewP for Honest vs. Deception responses. (**C**) Topographic voltage maps of the mean amplitude of the PCA-RewP. Error bars represent s.e.m. *p < 0.1, **p < 0.05.

The PCA-RewP (TF6/SF1) was quantified by the mean amplitude of the 50-ms window around the peak at Fz for each response, and a paired t-test was performed. The amplitude of PCA-RewP for the Honest response (mean ± se, 2.239 ± 0.306 μV) was significantly larger than that for the Deception response (mean ± se, 1.652 ± 0.279 μV, p = 0.031, effect size = 0.363, N = 38) (see Fig. 4).

#### Time-frequency domain analysis

**Figure 5:**
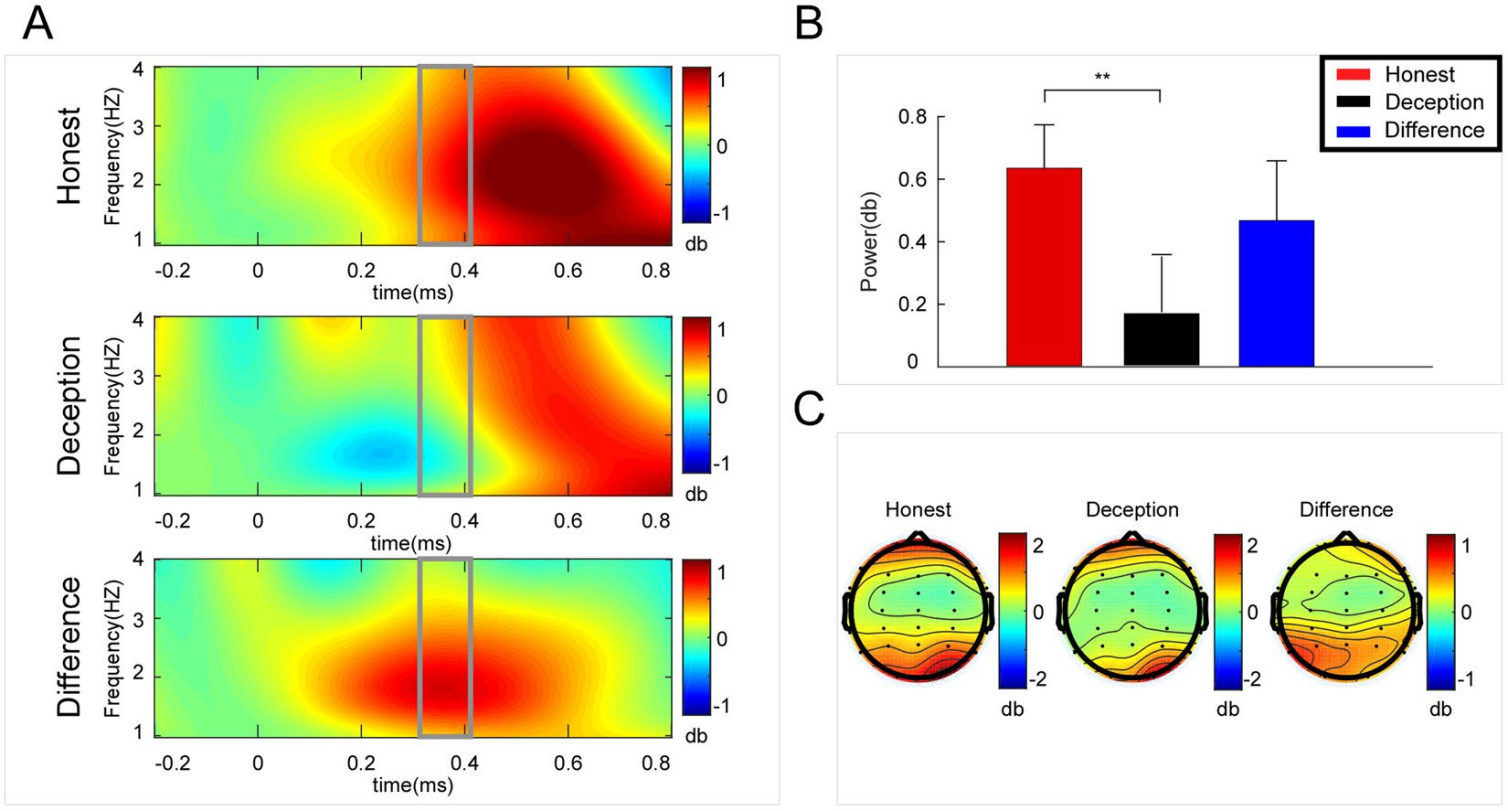
Delta results. (**A**) Delta (1–4 Hz) power changes at Pz for Honest vs. Deception responses. (**B**) Larger amplitude for the Honest response than for the Deception response. (**C**) Topographic maps of the mean amplitude of delta band power within 1–4 Hz from 360 to 410 ms. Error bars represent s.e.m. *p < 0.1, **p < 0.05.

**Figure 6:**
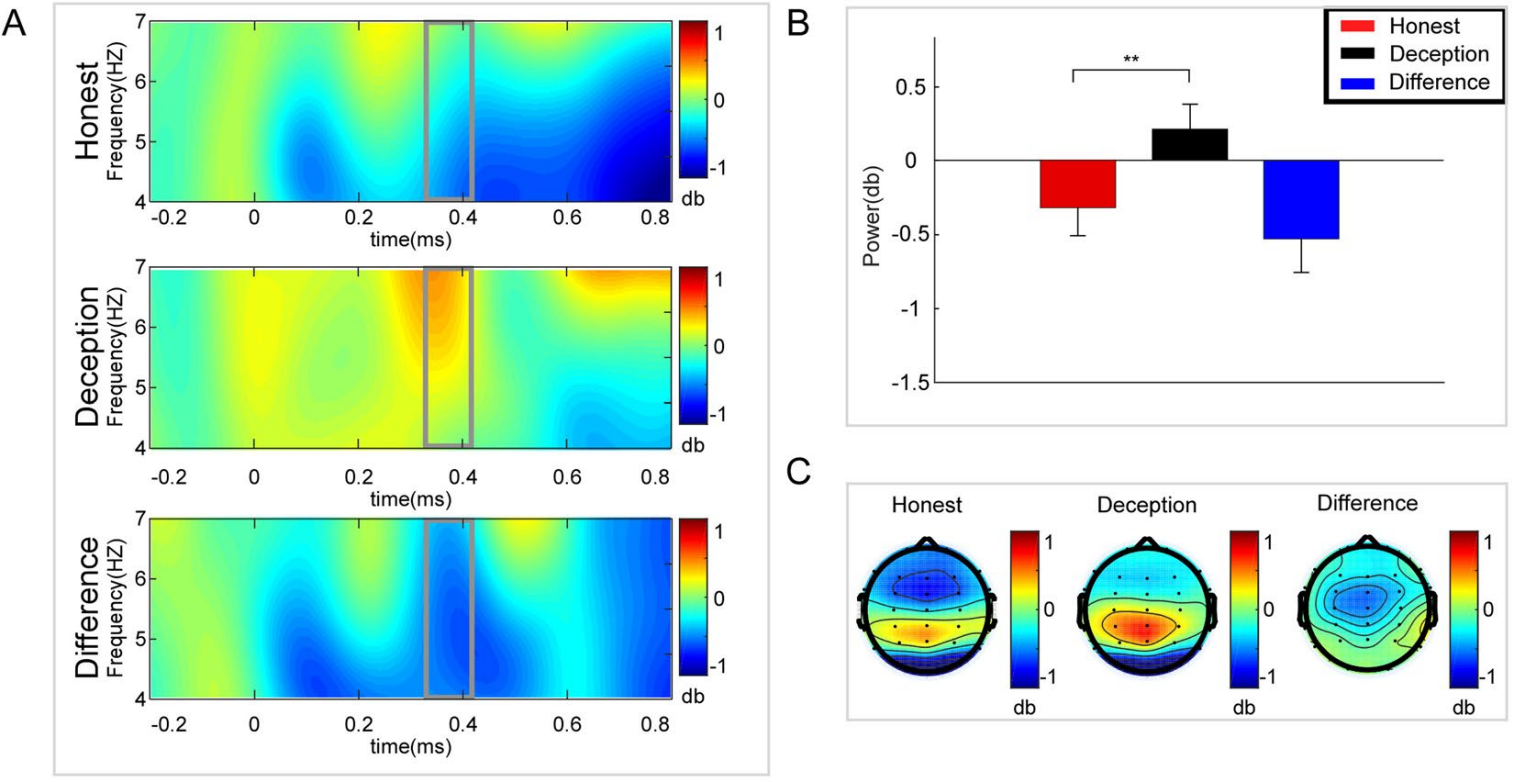
Theta results. (**A**) Theta (4–7 Hz) power changes at Cz for Honest vs. Deception responses. (**B**) Smaller amplitude for the Honest response than for the Deception response. (**C**) Topographic maps of the mean amplitude of theta band power within 4–7 Hz from 360 to 410 ms. Error bars represent s.e.m. *p < 0.1, **p < 0.05.

**Figure 7:**
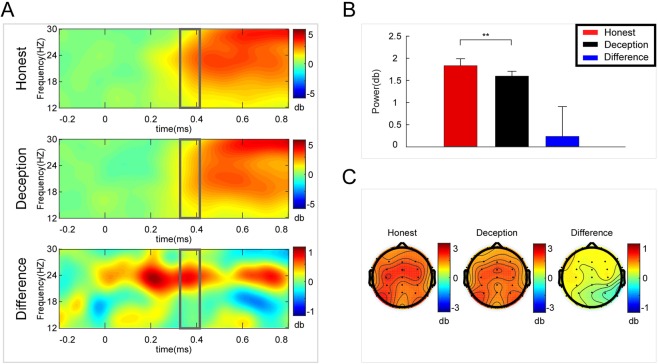
Beta results. (**A**) Beta (12–30 Hz) power changes at Fz for Honest vs. Deception responses. (**B**) Larger amplitude for the Honest response than for the Deception response. (**C**) Topographic maps of the mean amplitude of beta band power within 12–30 Hz from 360 to 410 ms. Error bars represent s.e.m. *p < 0.1, **p < 0.05.

A paired t-test was performed on the mean amplitude of delta band power (1–4 Hz) at Pz. The amplitude for the Honest response (mean ± se, 0.632 ± 0.139 dB) was significantly larger than that for the Deception response (mean ± se, 0.165 ± 0.189 dB, p = 0.019, effect size = 0.397, N = 38) (see Fig. 5). Then a paired t-test was performed on the mean amplitude of theta band power (4–7 Hz) at Cz. The amplitude for the Honest response (mean ± se, −0.318 ± 0.187 dB) was significantly smaller than that for the Deception response (mean ± se, 0.208 ± 0.166 dB, p = 0.024, effect size = −0.381, N = 38) (see Fig. 6). Moreover, a paired t-test was performed on the mean amplitude of beta band power (12–30 Hz) at Fz. The amplitude for the Honest response (mean ± se, 1.813 ± 0.136 dB) was significantly larger than that for the Deception response (mean ± se, 1.588 ± 0.666 dB, p = 0.045, effect size = 0.337, N = 38) (see Fig. 7).

## Discussion

Abundant behavioral and neurophysiological literature has studied the behavioral and neural correlates of spontaneous deception. However, few studies have focused on measuring the internal cost of spontaneous deception. In the present study, we used the ERP and ERSP techniques to measure the internal cost of spontaneous deception through a sender–receiver task in which participants decided whether to send deceptive messages to increase their payoff from the task. To our knowledge, our study is the first to investigate the internal cost of spontaneous deception. Several important main findings emerged from this study. In the time domain, we observed a RewP to be evident at 330 ms at FCz and a PCA-RewP at 340 ms at Fz after senders sent the message, suggesting an integration of reward with associated cost after response in our task. Spontaneous deception decreased the amplitude of the RewP and PCA-RewP, which are in response to reward evaluation, indicating that deceptive behavior carried an internal cost for individuals that devalued their rewards. Furthermore, in the time-frequency domain, spontaneous deception decreased the amplitude of power in the delta and beta bands but increased the amplitude of power in the theta band, supporting our finding regarding the time domain.

In line with a previous study, our behavioral data showed that several players were unwilling to lie even at the expense of earning extra money^3,5,6^. Moreover, our data demonstrated that deception rate is dependent on the payoff of truth-telling^4^. When the payoff was 20 CNY, senders truthfully revealed 17.23% of the messages. As the payoff increased, truthful reporting increased and reached 39.46% in the case of 25 CNY. Moreover, in the HI condition, in which the payoff was 30 CNY, the likelihood of truthful reporting was 97.98% and significantly higher than all other conditions. Thus, the higher the gain from lying was, the more frequent the deceptive behavior. This result validated that the decision to lie is dependent on the incentive.

We noted that for the HDI condition, the senders did not fully lie in approximately 2.39% sessions. The senders sent messages that earned them 25 CNY instead of 30 CNY. This finding is in line with a behavioral study^4^. Another type of deception, disadvantageous lying, was also found in our experiment. In the HI condition, approximately 2.02% of the messages were deceptive, which indicated that not all deceptive behaviors occurred for material gain. This type of deception was also found in a previous experiment^58^.

Not surprisingly, there is a substantial agreement that the deception in our task is socially inappropriate whereas the truth telling is socially appropriate. It seems that people believed that deception was improper and violated the social norm, even they did not know the other participant, and they would never meet this person. Since lying was believed against the social norm in our task, deceptively revealing messages might give rise to internal cost^3^. Therefore, when deciding whether to lie, participants might face an internal trade-off between honesty to avoid the potential internal cost of lying and dishonesty for personal material gain^4^.

Our behavioral data confirmed that the response time in the HDI condition was significantly longer than that in the HI condition. Given that response time is associated with the degree of cognitive conflict^59^, this result proved that individuals faced more cognitive conflicts when they made decisions in the HDI condition compared with the HI condition. Hence, when people had the incentive to lie, they needed to balance the material gain and internal cost of lying; thus, they faced further cognitive conflicts.

In the time domain, an ERP component (RewP) associated with reward evaluation and consumption was found at 330 ms at FCz after both honest and deception responses. The PCA technique used to disentangle overlapping ERPs extracted a pure RewP at 340 ms at Fz after response. The evidence of RewP after response suggested that there was an integration in reward processing after response. We examined the relation between deceptive behavior and the amplitude of the RewP. The ERP data illustrated that a larger RewP was elicited by the responses to send messages truthfully compared with the deceptive responses. Given that the RewP reflects the magnitude of hedonic pleasure experienced and the integrated reward^31,32^, the larger RewP elicited by truthful responses despite the same material gain indicated that deceptive behavior decreased the magnitude of hedonic pleasure experienced and the integrated reward per se. That is, deception carried an internal cost for individuals.

In the time-frequency domain, compared with deception, an honest response elicited a larger amplitude of power in the parietal delta band and the beta band at frontal midline sites, whereas it elicited a smaller amplitude of power in the theta band at the central midline. Recently, a number of studies confirmed that the delta band was the primary time-frequency component underlying the RewP and was sensitive to reward evaluation, leading to delta power being a reward-specific index of reward processing^31,44–49,60,61^. Similarly, the power in the beta band has also been linked to reward outcome. During reward processing, beta band power is sensitive to reward evaluation and reward magnitude^46,51,55^. In contrast to delta and beta band power, theta band power has been confirmed to be sensitive to loss^32,48–50^. Considering the changes in delta band power and beta band power, the results clearly showed that participants appeared to assign less value to the same payoff after lying than after truth-telling. Moreover, the power change in the theta band also suggested a loss from lying itself. These findings provide further evidence of the internal cost of deception.

The reward evaluation EEG components, namely, RewP, delta band power, beta band power and theta band power, were consistently elicited by feedback in previous studies^31,32,35,37,42,44–49^. However, the tasks in these studies, such as the door task, were somewhat different from ours. In these tasks, the participants did not know their reward until the outcome feedback occurred, whereas in our task, the participants knew their payoff as soon as they made the decision. Hence, the reward evaluation processing followed the responses in our task instead of feedback. This finding suggests that the RewP was essentially linked to the timepoint when participants knew their payoff but was not necessarily linked to the feedback. The reward evaluation EEG components are also sensitive to reward anticipation^46,62^. However, we presume that the participants in our task did not predict their payoff after response. The stimulus-preceding negativity (SPN), which appeared as a negative potential posterior to the response and peaked immediately prior to the presentation of outcome, was an index of reward anticipation^63,64^. The evidence that SPN was not found posterior to the response in our study supported our abovementioned presumption (See Supplementary Fig. S1). Therefore, the difference in the reward evaluation EEG components for honest vs. deception was not elicited by the participants’ predictions.

RewP was always suppressed by the NE in the previous door tasks^32^. Since the participants did not undergo the anticipatory phase after response in our task, we observed an obvious RewP after both honest and deception responses. In one of the previous studies, the RewP was eliminated when feedback was delayed^65^. These authors verified that the RewP integrated information about actions and outcomes. Moreover, it’s confirmed that the RewP integrated reward with associated cost anticipation and was a good candidate measure of the positive valence system construct of reward integration^31^. Hence, the RewP was enhanced by an honest response compared with a deception response, which indicated that reward was devalued by deceptive actions. In other words, deceptive actions carried an internal cost for the participants. Although the peak of our PCA-RewP occurred at 340 ms, which is inconsistent with other studies, its topographic voltage map and its location related to other PCA components (i.e., P2 and slow wave) are consistent with other studies^31,32^. One possible explanation for this latency delay in PCA-RewP was that reward integration processing started earlier after feedback than after response.

Taken together, our findings elucidated the unsolved issue of whether spontaneous deception carries an internal cost for individuals. Previous behavioral studies did not reach an agreement^3–5^. In the present study, we demonstrated that lying costs existed during spontaneous deception and rejected the self-concept maintenance hypothesis. Moreover, neuroscientific studies that focused on spontaneous deception asserted that self-control activity is associated with the decision to lie^19,21^. Individuals seemingly need to balance the gain and cost from lying in their decision to lie spontaneously^20^. In this regard, our finding was in line with the findings of these studies.

In sum, this study was the first to examine the existence of internal cost in spontaneous deception using the ERP and ERSP techniques. Behavioral results corroborated that deception rate and reaction time increased with the incentive. Based on the EEG data, deception elicited a small RewP, delta band power and beta band power, which were associated with reward evaluation and reward integration. Moreover, a larger theta band power, which was related to loss and cognitive effort, was elicited by deception. These results proved that deception carried an internal cost for the participants that decreased their experienced pleasure and integrated reward from the same payoff.

## Method

### Participants

A total of 45 healthy volunteers (mean age = 23.4 years; range = 21–25 years; female = 24) from Nankai University participated in this study for monetary compensation. All of their behavior data were used for behavior analysis. Seven subjects were excluded from the EEG analysis due to their inadequate number of deceptive trials because all participants behaved spontaneously. The brain activities of 38 subjects were fully analyzed. Sample size was determined based on the effect size of our experiment with 24 subjects in the first stage. All the participants were right-handed and native Chinese speakers. The participants had normal or corrected-to-normal vision and had no history of psychiatric or neurological disorders. Each participant signed written informed consent and received a base payment of 30 Chinese yuan (CNY, roughly equal to $4.50) for participation, plus a bonus of 20–30 CNY based on his/her decision. The study protocol was approved by the Ethics Committee of Nankai University. It was carried out in accordance with the approved guidelines and the declaration of Helsinki. Materials and data related to this experiment will be made available upon request.

### Stimuli and task

The players performed a sender–receiver task that was adopted from previous behavioral studies and modified for the current experiment^4^. In the experiment, the main unit of analysis was defined as a “trial,” in which two players were referred to as the “sender” and the “receiver”. All participants were assigned the role of sender and were informed that they would play the game with another player in the next room.

In each trial, three options (i.e., A, B, C) with different possible payoffs (i.e., 20, 25, 30) were shown on the screen. The computer then randomly assigned a letter A, B or C to the sender. Subsequently, the sender chose an option to send a message about this letter to the receiver. His/her message had to be one of the following: “The assigned letter is r”, and r was in accordance with his/her chosen option. Following the sender’s response, a black dot was presented. The outcome of that trial was then presented.

The sender’s payoff was in accordance with his/her chosen option and independent of the receiver’s decision. For example, if the sender chose option A, and the payoff for option A was 30, then he/she would earn 30 CNY for this trial. The receiver’s payoff was dependent on whether the letter he/she selected was the same letter as that assigned to the sender. If so, the receiver would earn 15 CNY for this trial; otherwise, he/she would earn 10 CNY. According to the payoff associated with the assigned letter, the sender faced three conditions, i.e., HI, LDI and HDI. In the HI condition, being honest would earn the sender 30 CNY, whereas disadvantageous lying, which is disadvantageous to the person in an individual decision problem, would earn the sender 20 or 25 CNY. In the LDI condition, being honest would earn the sender 25 CNY, whereas full lying, a deception maximizing one’s material gain, and disadvantageous lying would earn the sender 30 and 20 CNY, respectively. In the HDI condition, being honest would earn the sender 20 CNY, whereas partial lying, a deception without maximization of one’s material gain, and full lying would earn the sender 25 CNY and 30 CNY, respectively. With each condition containing 40 trials, a total of 120 experimental trials were performed. Prior to the experiment, the senders were told that one of the 120 trials would be selected to pay them after the experiment and were encouraged to use any strategy they wanted to maximize their bonus. Unbeknown to the senders, all the receivers were played by computers, and their choices were predetermined to be the same as the chosen options of the sender such that the receiver earned only 10 CNY when the senders lied (see Fig. 1).

After the experiment, all participants were asked to rate the extent to which sending a deceptive message or a true message in this task was socially appropriate. Each choice available to the sender in each condition was evaluated using 5-point Likert scale. A score of 1 represented a rating of “very socially inappropriate”, a score of 2 represented “somewhat socially inappropriate”, a score of 3 represented “not clear”, a score of 4 represented “somewhat socially appropriate”, and a score of 5 represented “very socially appropriate”.

### Procedure

EEG recording was conducted in a small, sound-attenuated, and electrically shielded chamber. After the EEG electrodes were attached, the participants sat in a comfortable chair that was approximately 100 cm in front of a 23-inch computer monitor. Before the tasks began, all the participants read the instructions carefully and were asked to take six practice trials. Figure 1A shows the timeline of a single trial. Each trial began with the presentation of a single centrally located red fixation varying from 400 to 800 ms. Thereafter, one of six possible combinations of options and payoffs (i.e., A.20, B.25, C.30; A.20, B.30, C.25; A.25, B.20, C.30; A.25, B.30, C.20; A.30, B.20, C.25; A.30, B.25, C.30) was shown on the screen for 600 ms. The order of these six combinations was counterbalanced. Afterward, a letter A, B or C appeared randomly. Subsequently, the senders were asked to send a message about this letter to the receivers, and feedback was presented for 1000 ms after a black dot, which varied from 2,000 to 2,500 ms.

The entire experiment comprised 120 test and six practice trials. Only the test trials were used for EEG analysis. The trials appeared in four blocks of 30 trials. Each block was separated by a break, the duration of which was determined by the senders. All 120 trials were performed within 15–25 min, during which the trials were randomly presented. E-Prime software was used to control the display of the stimuli and the acquisition of behavioral data (Version 2.0, Psychology Software Tools, Inc.).

### EEG acquisition

EEG data were recorded continuously with a 40-channel NuAmps DC amplifier (Compumedics Neuroscan, Inc., Charlotte, NC, USA). According to the International 10–20 System, 32 active Ag/AgCl electrodes were used. Electrodes below and above the left eye, as well as those located on the outer canthi of each eye, measured bipolar vertical and horizontal electrooculogram activities. EEG was sampled at 1000 Hz using a 22-bit A/D converter. The reference and ground electrodes were positioned at AFz, and the impedances of all electrodes were kept below 10 kΩ.

### EEG analysis

Preprocessing of EEG data was performed with EEGLAB 14.1.1^66^, implemented in MATLAB 2017a. A 1/30 Hz high-/low-pass filter was applied after the reference of EEG signals reset to the average of the left and right mastoids. Individual epochs were extracted from -1000 to 2000 ms around response. A manual artifact correction procedure was applied to eliminate trials with artifacts based on visual inspection. An independent component analysis (ICA) was run to remove eye movement, and the ICA components related to eye movement were manually selected. Artifact-free epochs of each subject were grouped into two conditions, i.e., Honest and Deception. To control the payoff effect, honest in the HI condition and full lying in the HDI condition were used to represent Honest and Deception, respectively. For these two conditions, the same number of trials (i.e., 30 trials) was used (randomly resampled) based on the condition with the lowest trial count. Finally, ERPs and ERSPs were based on 60 trials per participants.

Clean EEG data were analyzed in the time domain. The 1000-ms epochs were extracted starting at 200 ms before the response of the sender’s decisions. A 200-ms preresponse period was used as baseline, and the accepted epochs were baseline-corrected. The RewP was scored as the mean voltage from 305 to 355 ms postresponse at a set of midline sites (FCz, Cz) corresponding to the 50-ms time window surrounding the peak (see Fig. 2). Moreover, a temporospatial PCA was used to extract the RewP. PCA was conducted with the EP Toolkit (v2.45) for MATLAB following the two-step procedure. A temporal PCA was performed first using promax rotation, and 18 temporal factors were extracted based on the scree plot. A spatial PCA was then performed on each temporal PCA using infomax rotation, and 2 spatial factors were extracted, yielding 36 factor combinations. Based on visual inspection of grand average waveforms, PCA-RewP (TF6/SF1) was chosen for further statistical analyses.

Time-frequency analysis was performed using the Fieldtrip toolbox built-in ft_freqanalysis () function, based on complex Morlet wavelet convolution (2–10 cycles, 1–30 Hz, 120 spaced frequencies, 1000 time points per epoch)^67^. The time interval −200 to 0 ms before response was used for baseline normalization. Based on visual inspection, the mean converted amplitude within 1–4 Hz from 360 to 410 ms at Pz was used to analyze delta band power change. The same approach was adopted for the analysis of theta and beta band power changes. The mean converted amplitude within 4–7 Hz from 360 to 410 ms at Cz and within 12–30 Hz from 360 to 410 ms at Fz were used to analyze theta and beta band power changes, respectively.

For all analyses, p-values were corrected using the Greenhouse–Geisser correction when the sphericity assumption was violated. P < 0.05 was considered significant, and p < 0.1 was considered marginally significant. Significant interaction was analyzed using the simple effect model. Statistics were analyzed using IBM SPSS 19.0 software.

## Supplementary information

Internal cost of spontaneous deception revealed by ERPs and EEG spectral perturbations

## Data Availability

The datasets generated during the current study are available in the 4TU.ResearchData repository, 10.4121/uuid:c9609050-bc50-451a-a9d0-b622f4c12927.
