# Supplementary material for: Internal cost of spontaneous deception revealed by ERPs and EEG spectral perturbations
Source: Sci Rep. 2019 Apr 1;9:5402. doi: 10.1038/s41598-019-41962-z (PMC6443694; doi:10.1038/s41598-019-41962-z)
Supplement: Supplementary file 1 — Internal cost of spontaneous deception revealed by ERPs and EEG spectral perturbations [file 41598_2019_41962_MOESM1_ESM.docx]

**Supplementary Information**

**Internal cost of spontaneous deception revealed by ERPs and EEG spectral perturbations**

Chengkang Zhu^1,3^, Jingjing Pan^1,3^, Shuaiqi Li^1,3^, Xiaoli Liu^1,3^, Pengcheng Wang^3,5^, Jianbiao Li^2,3,4*^

1 China Academy of Corporate Governance, Business School, Nankai University, Tianjin, China

2 School of Economics, Shandong University

3 Reinhard Selten Laboratory, Nankai University, Tianjin, China

4 Nankai University Binhai College, Tianjin, China

5 Business School, Tianjin University of Economic and Finance, Tianjin, China

Chengkang Zhu and Jingjing Pan contributed equally to this work

Correspondence and requests for materials should be addressed to J.L. (email: biaojl@126.com)

| **Supplementary Table 1. Social appropriateness rating** | Very socially appropriate (%) | 66.67 | 0 | 2.22 | 6.67 | 57.78 | 0 | 2.22 | 2.22 | 62.22 |
| --- | --- | --- | --- | --- | --- | --- | --- | --- | --- | --- |
|  | Somewhat socially appropriate (%) | 28.89 | 11.11 | 8.89 | 11.11 | 40 | 13.33 | 11.11 | 8.89 | 26.67 |
|  | Not clear (%) | 0 | 0 | 0 | 2.22 | 0 | 0 | 8.89 | 8.89 | 8.89 |
|  | Somewhat socially inappropriate (%) | 0 | 75.56 | 55.56 | 40 | 2.22 | 46.67 | 31.11 | 46.67 | 0 |
|  | Very socially inappropriate (%) | 4.44 | 13.33 | 33.33 | 40 | 0 | 40 | 46.67 | 33.33 | 2.22 |
|  | Sender’s payoff | 20 | 25 | 30 | 20 | 25 | 30 | 20 | 25 | 30 |
|  | Response | Truth-telling | Lying | Lying | Lying | Truth-telling | Lying | Lying | Lying | Truth-telling |
|  | Condition（n=45） | HDI | | | LDI | | | HI | | |


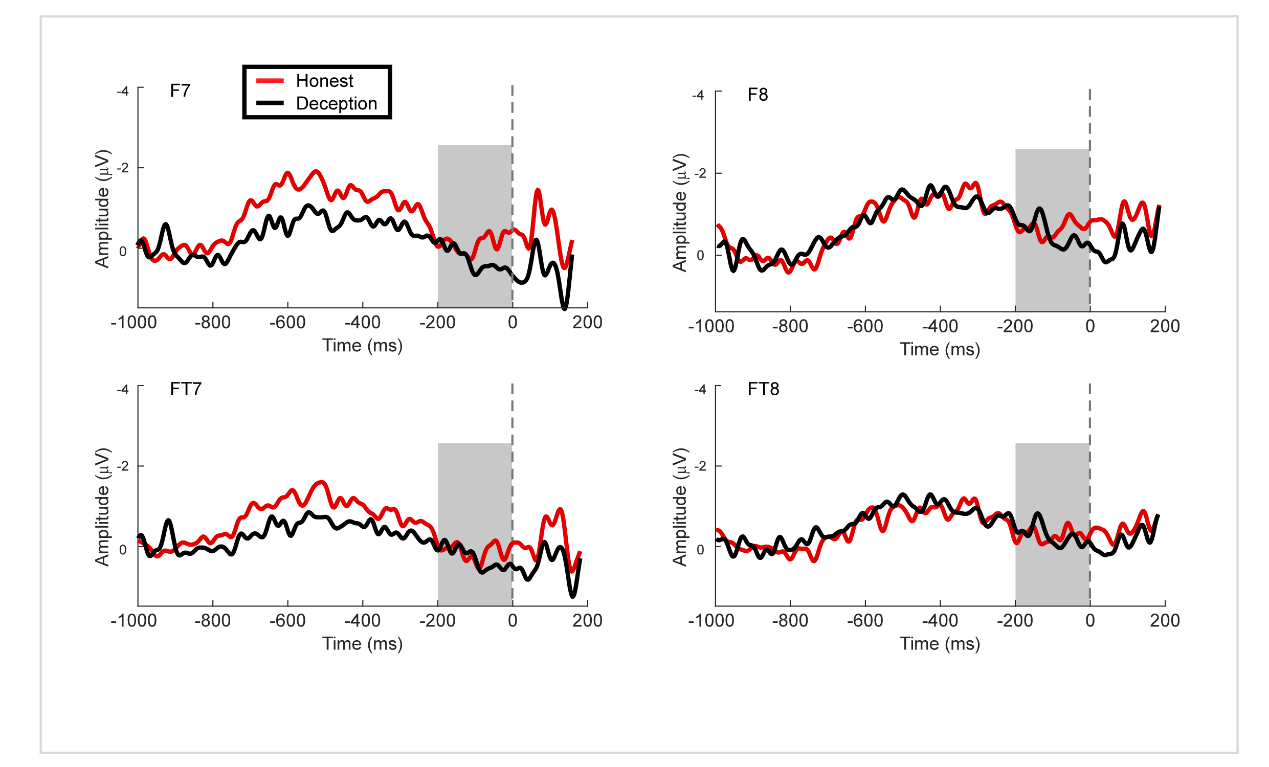
Supplementary Figure S1. SPN waveform. Grand average ERP waves prior to feedback at F7, F8, FT7 and FT8.
